# A Clinical Trial to Increase Self-Monitoring of Physical Activity and Eating Behaviors Among Adolescents: Protocol for the ImPACT Feasibility Study

**DOI:** 10.2196/18098

**Published:** 2020-06-05

**Authors:** Justin B Moore, Joshua R Dilley, Camelia R Singletary, Joseph A Skelton, David P Miller Jr, Vahé Heboyan, Gianluca De Leo, Gabrielle Turner-McGrievy, Matthew McGrievy, Edward H Ip

**Affiliations:** 1 Department of Implementation Science Division of Public Health Sciences Wake Forest School of Medicine Winston-Salem, NC United States; 2 Department of Plastic Surgery Wake Forest School of Medicine Winston-Salem, NC United States; 3 Department of Pediatrics Wake Forest School of Medicine Winston-Salem, NC United States; 4 Department of Internal Medicine Wake Forest School of Medicine Winston-Salem, NC United States; 5 Department of Interdisciplinary Health Sciences Augusta University Augusta, GA United States; 6 Department of Health Promotion, Education & Behavior Arnold School of Public Health University of South Carolina Columbia, SC United States; 7 Office of Operations and Accreditation Arnold School of Public Health University of South Carolina Columbia, SC United States; 8 Department of Biostatistics & Data Science Division of Public Health Sciences Wake Forest School of Medicine Winston-Salem, NC United States

**Keywords:** physical activity, obesity, adolescence, adult, therapy, family, mobile phone

## Abstract

**Background:**

Severe obesity among youths (BMI for age≥120th percentile) has been steadily increasing. The home environment and parental behavioral modeling are two of the strongest predictors of child weight loss during weight loss interventions, which highlights that a family-based treatment approach is warranted. This strategy has been successful in our existing evidence-based pediatric weight management program, Brenner Families in Training (Brenner FIT). However, this program relies on face-to-face encounters, which are limited by the time constraints of the families enrolled in treatment.

**Objective:**

This study aims to refine and test a tailored suite of mobile health (mHealth) components to augment an existing evidence-based pediatric weight management program.

**Methods:**

Study outcomes will include acceptability from a patient and clinical staff perspective, feasibility, and economic costs relative to the established weight management protocol alone (ie, Brenner FIT vs Brenner FIT + mHealth [Brenner *m*FIT]). The Brenner *m*FIT intervention will consist of 6 mHealth components designed to increase patient and caregiver exposure to Brenner FIT programmatic content including the following: (1) a mobile-enabled website, (2) dietary and physical activity tracking, (3) caregiver podcasts (n=12), (4) animated videos (n=6) for adolescent patients, (5) interactive messaging, and (6) in-person tailored clinical feedback provided based on a web-based dashboard. For the study, 80 youths with obesity (aged 13-18 years) and caregiver dyads will be randomized to Brenner FIT or Brenner *m*FIT. All participants will complete baseline measures before randomization and at 3- and 6-month follow-up points.

**Results:**

This study was approved by the Institutional Review Board in July 2019, funded in August 2019, and will commence enrollment in April 2020. The results of the study are expected to be published in the fall/winter of 2021.

**Conclusions:**

The results of this study will be used to inform a large-scale implementation-effectiveness clinical trial.

**International Registered Report Identifier (IRRID):**

PRR1-10.2196/18098

## Introduction

Severe obesity among youths (BMI for age ≥120th percentile) has been steadily increasing. Declines in physical activity over the last four decades have been accompanied by concurrent declines in dietary quality [1], increased sugar consumption [2], and increased energy intake [3]. The combination of these factors has contributed to the increased prevalence of overweight/obesity in youths during this period, particularly severe childhood obesity [4]. Clinical interventions to treat obesity through healthy eating and physical activity show that improvements in these behaviors are linked to modest, positive weight outcomes [5] and improvement in cardiometabolic risk factors [6]. However, pediatric weight loss programs have relied on in-person visits, creating difficulties for parents’ work schedules and the need to travel. A US Preventive Task Force panel noted that intensive clinical programs are effective but require ≥26 hours of contact to achieve positive results, which is difficult for the youths and their families to do [7]. Mobile technology supporting health practices (mobile health [mHealth]) can reduce these barriers by delivering theoretically based content, facilitating self-monitoring, and connecting families to enhance treatment adherence and improve weight status [5].

Involving family members in the treatment of adolescents with obesity can be effective, but adherence is suboptimal [6]. Few studies have targeted youths and their families with an mHealth approach to supplement a clinical weight loss intervention [8] despite evidence for its acceptability and feasibility in adolescents and adults [8,9]. Parent-child interventions are more effective than treatment programs that focus primarily on the child, and mHealth strategies are ideally suited for intervention targets identified in previous conceptual models (eg, increasing parental competence, self-efficacy, parent-child communication, and parents’ support of the adolescent’s autonomy) [10]. Engaging parents or other caregivers in treatment plans can be challenging, but mHealth strategies can potentially increase the caregivers’ exposure to and engagement with intervention strategies by providing caregivers with more easily implemented tools to monitor youth behaviors and facilitate communication between caregivers and adolescents.

Self-monitoring is the cornerstone of behavioral treatment for weight loss [11], and both adherence to self-monitoring [12] and tailored feedback on self-monitoring behaviors [13] are associated with weight loss. mHealth technologies can be used to deliver the behavioral content of a weight loss intervention and provide participants with ways to self-monitor and receive feedback. Moreover, mHealth can deliver efficacious approaches for weight loss as phone ownership is pervasive in both adults [14] and children [15]. mHealth has the potential to make monitoring of healthy eating and physical activity less burdensome, although limited tools exist for parents and children. Therefore, there is an opportunity to develop and test mHealth tools to augment clinical practices. This paper describes the study design and conceptual approach of developing mHealth tools that will then be formally evaluated in a full-scale randomized trial to assess their effectiveness. The current project will randomize 80 youths with obesity (aged 13-18 years) and caregiver dyads to a pediatric weight management program or the same program plus a suite of mHealth tools. This study will establish the acceptability, feasibility, and costs of the mHealth program relative to the standard of care (ie, family-based, multidisciplinary weight management), and the proposed sample of 80 dyads will give sufficient power for the estimation of retention rates and confidence intervals to inform a large-scale implementation-effectiveness clinical trial (see Trial Sample Size section).

## Methods

### Study Setting

Brenner Children’s Hospital is a 160-bed children’s hospital, which is part of the Wake Forest Baptist Medical Center (WFBMC). Located at the WFBMC, Brenner Families in Training (Brenner FIT) is an interdisciplinary, family-based pediatric weight management program [16-18] that focuses on the treatment of obesity in children aged 2 to 18 years. A physician referral is required, and treatment involves the entire family. The Brenner FIT team includes physicians, family counselors, dietitians, social workers, an activity/play specialist, and a physical therapist. Brenner FIT materials are available in English and Spanish. Brenner FIT also offers free nutrition and face-to-face parenting classes to all members of the community. Given that Brenner FIT provides care for a diverse patient population, it will serve as the base of the study. The average patient is clinically obese, as indicated by a BMI of 35.9 kg/m^2^ (SD 8.6) and a BMI z-score of 2.6 (SD 0.5). Half of the patient population is in their teenage years (13-18 years). Brenner FIT is successful in enrolling a diverse population of families into research studies [19,20]. Approximately 66% of those enrolled in Brenner FIT improve their weight status [21], and the children who are successful display a decrease in BMI z-score of 0.07 to 0.1, with an average BMI z-score decrease of 0.11 at 8 months. A 0.1 to 0.15 decrease in BMI z-score is linked with healthy changes in cardiometabolic biomarkers [21].

### Study Design: Inclusion/Exclusion Criteria and Recruitment

Caregiver/adolescent dyads referred to the Brenner FIT program are invited to participate. Dyads are eligible if the adolescent under treatment is aged 13 to 18 years, a caregiver who lives in the house (eg, a parent or grandparent) agrees to participate, both members of the dyad (the adolescent and caregiver) own a smartphone or tablet, and the adolescent has no contraindications for physical activity as indicated by their physician. Research suggests that smartphone ownership is greater than 95% in the target population [22]. The youth will be excluded from participation in the study if they have functional limitations that preclude engaging in physical activity as directed by the program because of our inability to tailor the mHealth materials for those requiring adaptations. Those deemed eligible initially are informed of the study following their intake visit by Brenner FIT clinical staff. Those who express interest receive an in-person *warm handoff* to a research team member who confirms eligibility, reassesses interest, and performs or schedules an intake visit.

### Informed Consent and Intake

Following the clinical visit, caregivers and patients are informed of the study and are invited to learn more from the research staff. At this orientation session, the research staff describe the study and administer consent/assent forms to those who wish to enroll. Participants complete the consent/assent forms, then complete all baseline survey measures, have their weight/height assessed, and are oriented to the procedure for physical activity measurement via an accelerometer. ActiGraph accelerometers are affixed to all youth participants via a wrist strap, and the family is given a self-addressed, padded envelope to return the accelerometer in after 7 days of wear. Once participants return the monitor and complete dietary assessments, they are contacted to learn their group assignment (ie, Brenner FIT or Brenner FIT + mHealth [Brenner *m*FIT]). Follow-up visits are scheduled by the clinical staff in coordination with the research staff who complete assessments at 3 months (a subset of measures), and at 6 months, with the participating dyads following their clinical appointments at both time points.

### Study Design: Randomization

This study is registered in ClinicalTrials.gov (NCT03961061). The study will be a two-group, randomized controlled superiority trial design. Dyads will be block randomized by our statistician to 1 of 2 groups. The 2 groups are (1) Brenner FIT (standard care: n=40 dyads) and (2) Brenner *m*FIT (standard care plus mHealth features: n=40 dyads). Owing to the nature of the study, it is impossible to blind the clinical staff or participants, but all data will be collected by an independent evaluator who is blind to the condition and not involved in the delivery of the intervention.

### Control Condition: Brenner Families in Training (Standard Care)

Brenner FIT is an interdisciplinary, family-based pediatric weight management clinic. Treatment teams comprised a pediatrician, counselor, dietitian, and physical activity specialist, with others (eg, social workers, physical therapists) as needed. The entire family is encouraged to attend all aspects of the treatment program, although only 1 caregiver is required. As recommended with tertiary care stage 3 to 4 weight management programs [23], treatment is guided by an established protocol that is monitored using a clinical database.

After referral to Brenner FIT, families attend an orientation, following which they are scheduled for an initial introductory 2-hour intake group session; these occur within 2 to 4 weeks of the orientation. Monthly 1-hour-long visits with the dietitian, counselor, and physical activity specialist are held for 6 months, after which the child and caregiver see the pediatrician. During the 6 months of treatment, they attend 4 group classes, choosing from topics such as meal planning, physical activity, and parenting. Specialized visits with the physical activity specialist or physical therapist are scheduled when pertinent issues arise. Clinic visits include individualized goal setting (for behaviors that the family/clinician have jointly agreed to address), healthy eating and physical activity education, and behavioral counseling to implement changes at home. Motivational interviewing, modified by Brenner FIT for use with families [24], is key to treatment; family counselors are trained in cognitive behavioral therapy as well as parenting support and education, nonrestrictive approaches to dietary modification, and mindfulness and employ these approaches to assist families in developing healthy habits. Self-monitoring is part of the intervention: parents and children complete handwritten paper diet and physical activity logs and return them to clinicians.

#### Intervention Condition: Brenner Families in Training Plus Mobile Health

Brenner *m*FIT includes all components of the standard Brenner FIT program and 6 mHealth components that are designed to target theoretically supported constructs (Table 1). The 6 mHealth components are (1) a mobile-enabled website, (2) dietary and physical activity tracking and a physical activity tracker, (3) caregiver podcasts (n=12), (4) animated videos (n=6) for adolescent patients, (5) interactive messaging (between the participants and clinical staff), and (6) tailored clinical feedback. All Brenner *m*FIT components are delivered by the clinical staff. The components are outlined individually as follows.

**Table 1:** Components of the Brenner Families in Training plus Mobile Health condition and associated theoretical constructs.

Component	Use/Content	Theoretical construct
Mobile-enabled website	The mobile-enabled website will serve as the central hub of the mHealth components and will enable easy access to study materials and feedback	Facilitation (SCT^a^)
Diet and physical activity tracking	Tracking physical activity via the Fitbit wearable and behavioral food goals tracked via the mobile-enabled website	Facilitation (SCT); self-regulation (SCT)
Caregiver podcasts	A 12-part episodic story about caregivers who are working with their adolescent children to help them to be active, eat healthy, and achieve a healthy weight. The podcasts will deliver theoretically informed content around emotional regulation, proper use of incentives, and providing support for their child to gain autonomy over their weight loss program	Outcome expectations (SCT); self-efficacy (SCT); incentive motivation (SCT); autonomy support (SDT^b^)
Youth-animated videos	A 6-part series of short videos about teenagers who are struggling but succeeding with commonly encountered challenges to weight loss. Each episode will take on a different scenario that youth face successfully. The brief videos will use a compelling story and humor to address tough situations that the youths may find themselves in, such as navigating the holidays, being active despite barriers (eg, rain, inactive friends), shopping/cooking for oneself, or lack of motivation to be active	Observational learning (SCT); outcome expectations (SCT); autonomy (SDT); relatedness (SDT)
Peer and professional support	Secure platforms will be created so that caregivers can give and receive encouragement and feedback from peers and the clinical staff. The clinical staff will also give direct feedback regarding goal progress via directs texts and emails with families based on personalized reports provided to the clinical staff	Relatedness (SDT); self-efficacy (SCT)
Tailored clinical feedback	Personalized reports from each dyad will be provided to the clinical team on a weekly basis, which will give insight into successes and challenges related to self-monitoring, goal setting, goal attainment, and engagement with intervention materials	N/A^c^

^a^SCT: social cognitive theory.

^b^SDT: self-determination theory.

^c^N/A: not applicable.

#### Component 1: A Mobile-Enabled Website

A mobile-enabled website (Multimedia Appendix 1) developed for the project and accessible via a telephone, tablet, or personal computer serves as a central hub for the materials. Podcasts, animated videos, tracking summaries (for the parent and child), goal setting, and messages are delivered via the website utilizing an application program interface that integrates data from a commercial app (Fitbit) with data entered by participants.

#### Component 2: Dietary and Physical Activity Tracking

A wearable tracking device, chosen by the study team after consultation with caregivers and patients from previous work, is utilized for physical activity self-monitoring. Caregivers and adolescents are instructed to download the Fitbit app to their mobile devices, and adolescents are given a Fitbit Inspire HR activity tracker which synchronizes with the Fitbit app. Mobile phone apps have been shown to facilitate increased self-monitoring [25], which supports tracking of progress on behavioral goals (a component of Brenner FIT) by the youth and reporting to the caregiver and clinical team. Tracking app data are integrated into the mobile-enabled website.

Dietary behavioral tracking is supported by the mobile-enabled website. This strategy was chosen over the use of commercially available apps because no commercially available apps are congruent with the goals of the Brenner FIT program. The mobile-enabled website lets caregivers and youths track their goals in a manner that is appropriate for their role in the weight loss journey. For example, parents are asked to set goals related to providing a healthy food environment (eg, provide 5 family dinners this week), whereas youths are asked to set goals related to their behaviors (eg, eat 5 dinners with my family).

#### Component 3: Caregiver Podcasts

Caregiver podcasts tell a story of a caregiver of an adolescent Brenner FIT patient helping his/her child achieve a healthy weight. Through an engaging story, podcasts will help caregivers deal with the emotions that come with raising an adolescent in treatment for a medical condition (ie, obesity), provide strategies and encouragement to caregivers to help their adolescent build autonomy for healthy behaviors, engage in age-/ability-appropriate physical activity with their child, cook healthy family meals, and provide healthy snacks. Podcasts additionally focus on positive communication, interactions, and challenges often encountered in parenting. Podcasts have been used successfully in 4 previous studies by the team [26-30] and target several constructs from the self-determination theory and social cognitive theory, including (1) outcome expectations, (2) self-efficacy, (3) incentive motivation, and (4) autonomy support. Podcasts will be 5 to 10 mins long each and downloadable via the mobile-enabled website. Caregivers can download and listen to 1 podcast each week in weeks 1 to 12.

#### Component 4: Animated Videos

Animated videos were created based on feedback from adolescents previously enrolled in the Brenner FIT program. In this study, youths are given access to these animated episodic videos in six 30-second episodes. Animated videos are available to the youth biweekly for the first 12 weeks. Animated videos introduce scenarios encountered by adolescents who are part of a program like Brenner FIT. Each episode follows the youths as they deal with the negative and positive emotions of their weight loss journey and the difficult social situations in which they find themselves engaged. The stories and scenarios contain elements of humor and drama while targeting several constructs from the self-determination theory and social cognitive theory, including (1) observational learning, (2) outcome expectations, (3) autonomy, and (4) relatedness. Similar to the podcasts, the videos are accessible from the mobile-enabled website. The scenarios were developed based on feedback provided by the clinical staff with input from a panel of families enrolled in the Brenner FIT program. Audio for the videos is available in English with subtitles available in English and Spanish.

#### Component 5: Interactive Messaging

Peer support is associated with weight loss [31]. Therefore, opportunities are created for peer social support among caregivers and youths on the mobile-enabled website. As many adults do not have social media accounts, we also created a private, secure, moderated message board on the mobile-enabled website. Participants (caregivers and adolescents) are encouraged to (1) post questions to the clinical staff, (2) participate in group discussions and challenges (eg, *post a picture of your healthiest meal today*), (3) post about physical activities, and (4) share their success stories and challenges. Participants on social media are encouraged to follow each other as well as a curated list of health professionals on social media.

#### Component 6: Tailored Clinical Feedback

Tailored feedback is provided at regularly scheduled clinic visits by the clinical staff based on a web-based dashboard generated from self-monitoring data. The clinical staff have access to the web-based dashboard that provides a summary of the level of self-monitoring, level of physical activity, and progress on behavioral/weight loss goals for the adolescent and their caregiver. The staff use this information to engage with dyads via regularly scheduled face-to-face meetings and emails to give feedback based on behavioral progress, encouragement to engage in greater self-monitoring, and/or theoretically informed messages to promote self-efficacy, positive outcome expectations, and self-regulatory behaviors.

### Primary Outcomes

#### Primary Aim: Pilot the Intervention in Dyads Recruited From a Pediatric Weight Loss Clinic to Establish Acceptability and Feasibility of the Intervention Relative to Standard Care

Acceptability is measured using a brief survey from caregivers and youths to gain insight into their reception of the 6 mHealth components following the completion of the 6-month assessment (ie, completion of the study). Surveys include the system usability scale [32] to inquire about the usability of the mobile-enabled website, tracking apps, Fitbit tracking device, podcast format and content, animated video format and content, and web-based interactions with other participants and the clinical staff.

Feasibility is assessed consistent with the recommendations of Leon et al [33]. Specifically, the team examines screening, recruitment, randomization, retention, adherence, fidelity, and the assessment process. The number of patients screened per month, the number enrolled per month, the proportion of those enrolled who are eligible, and the number who remain enrolled in the study by condition are all tracked. Retention rates (completion of at least 75% of monthly sessions through 6 months) are tracked for each intervention condition. The time needed for assessments is monitored, and feedback on participant burden is recorded. Examples of clinical staff data include increases in clinical staff time interacting with patients and families, costs of delivering clinical staff training (eg, orientation to the technology), time spent in technical support with families related to mHealth components, and hosting/maintenance of the mobile-enabled website. The team developed a brief survey for the clinical staff to gain insight into the benefits and challenges of using the mHealth components and suggestions for improvements. Previous publication documents from the study group have demonstrated good engagement, retention, feasibility, and acceptability of a mobile tracking intervention in youths and their families [34].

**Figure 1:**
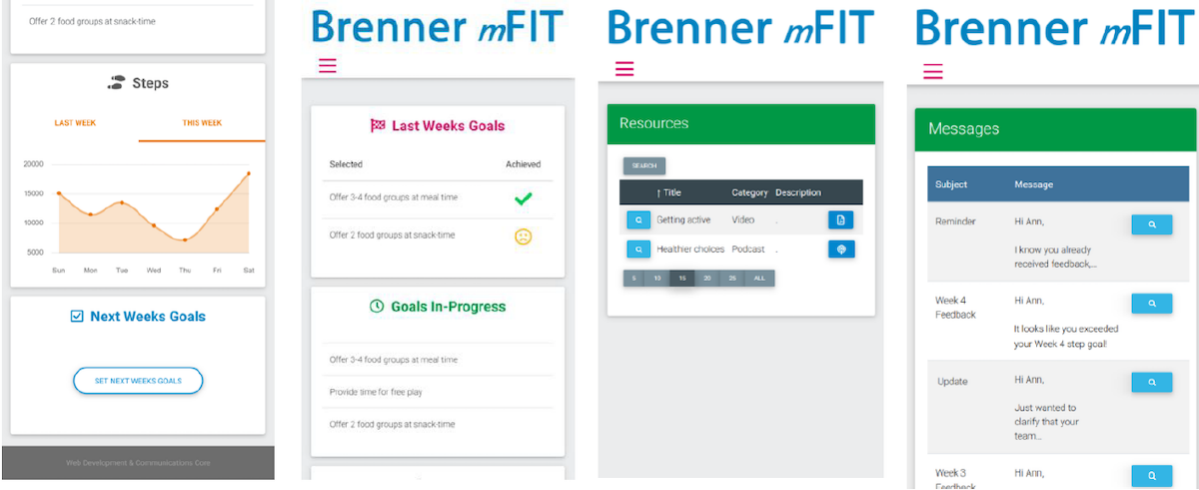
Screenshots showing (left to right) physical activity monitoring, goal setting, resources, and message board.

Implementation fidelity is assessed to inform a future implementation-effectiveness study. Specifically, activity by the clinical staff and participants is compared against what has been directed (staff) or prescribed (participants). For example, the number of downloads of podcasts/videos, frequency of dietary and physical activity self-monitoring, goal setting, and open rates for web-based messaging from the clinical staff are recorded (Figure 1). The logs of participant engagement with website components are utilized rather than using self-reporting to remove recall bias. In addition, the participant retention rate is considered as a measure of fidelity (the criterion for retention rate has been detailed in the Trial Sample Size section). These data give indicators of fidelity and dose and highlight areas for improvement in future studies.

### Procedures and Measures

#### Youth Measures Only: Physical Activity and Diet

Accelerometer data are collected at a 7-day interval at baseline and again at 6 months using ActiGraph GT3X+ accelerometers. The accelerometers are set to collect data in the *raw* format and analyzed by converting the data to 1-second epochs to account for the intermittent and sporadic nature of children’s physical activity [35] and to improve the ability to capture the transitory physical activity patterns of children. During each 7-day data collection period, these monitors are affixed to a wrist strap on the participant’s nondominant hand. Research by our team and others suggest that compliance is greater when the accelerometer is worn on the wrist, and this placement also allows for comparison with the National Health and Nutrition Examination Survey normative data collected at the wrist beginning in 2011 [36-38]. The cut points are distilled in a manner consistent with emerging research that allows for comparability with waist-based estimates [39]. Participants are instructed to wear the monitor continuously over the next 7 days except during bathing and sleeping. Once an accelerometer is received, data from the accelerometer are downloaded using the manufacturer’s software and a USB computer interface. The accelerometer data are reduced, cleaned, and processed using custom scripts in Stata (StataCorp). The accelerometers are initialized and set to record beginning at 5 AM the day following their distribution. This provides 7 days of data for analysis, from which 4 or more complete (>10 hours) days will be extracted. Participant’s accelerometer data are included in the analyses if 4 days with 10 hours of data are available.

Diet is assessed using the National Cancer Institutes’ automated self-administered 24-hour (ASA24) dietary assessment tool (version: ASA24-2016) on 3 nonconsecutive days (including 1 weekend day). The ASA24-2016 is available on both computers and mobile devices and takes approximately 25 min to complete. All youth participants complete one ASA24 at their baseline orientation and at 6-month follow-up office visits to encourage compliance while allowing youths and caregivers to ask questions and gain comfort with using the system. The project coordinator contacts participants within 6 days (on a randomly selected day) following the office visits to inform participants that they should complete the second/third dietary recall and offers to conduct the recall over the phone if the youths need assistance (the ASA24 has been shown to perform equally well as a self-administered or interviewer-administered questionnaire) [40,41]. A series of follow-up reminder messages and/or phone calls are made by the coordinator to encourage completion of the second/third of the two recalls if not completed within 24-hours following the first contact after the clinic visit. Dietary data are collected at baseline and 6-month follow-up.

#### Caregiver and Youth Measures: Psychosocial Variables, Sociodemographic Variables, and Weight Status

Family and individual (youth and caregiver) constructs are assessed to identify potential mediators or moderators of observed effects of the intervention; these are incorporated into a larger trial in the future. Brenner FIT uses the family systems theory as a guiding model to address child and parent behaviors within the context of their family [42] and presently uses a number of scales to assess families participating in Brenner FIT. Specifically, Brenner FIT families complete the Family Assessment Device General Functioning subscale (capturing family function) [43], Olson’s Family Communication Scale [44], perceived stress [45,46], self-efficacy for physical activity, impulsivity, and health behaviors of the family [47]. In addition, the team administers scales to capture social cognitive theory and self-determination theory constructs targeted by the intervention that are not captured as part of the intake process. These include outcome expectations, autonomy, autonomy support, and relatedness [48]. All psychosocial data are collected at baseline (before randomization), at 3 months, and at 6 months. Details regarding the variables collected, their theoretical rationale, and measurement instrument are presented in Table 2.

The caregiver and youth participants’ age, sex, race, and ethnicity are collected via a parental self-report upon enrollment in Brenner FIT. The youth participants will also self-report their gender, race, and ethnicity during baseline data collection. The weight status of caregivers and youths are quantified through the calculation of BMI derived from the measurement of height and weight at the intake and follow-up visits. Both height (SD 0.1 cm) and weight (SD 0.5 kg) are recorded twice, and the values are averaged to produce the final value. BMI is calculated as kg/m^2^. Height and weight are measured without shoes in normal clothing. The BMI z-score and percent of the 95th percentile BMI are calculated using the Centers for Disease Control and Prevention growth charts.

**Table 2:** Intervention-targeted constructs to be measured, participant providing data, theory, and instrument or method used.

Construct	C/Y^a^	Theory	Instrument or method
Weight status	Y	N/A^b^	Measured height and weight: used to calculate the BMI (weight in kg/height in m^2^) and calculate the BMI z-score and percent over the 95th percentile
Physical activity	Y	N/A	Accelerometry (7 days of monitoring): used to estimate minutes of moderate-to-vigorous physical activity per day
Dietary intake	Y	N/A	Automated self-administered 24-hour (ASA24-2016) dietary assessment tool: used to estimate dietary composition
Perceived autonomy support	Y	SDT^c^	Perceived parental autonomy support scale: produces a continuous score
Autonomy support	C	SDT	Motivators’ Orientations Questionnaire: produces a continuous score
Impulsivity	C	SDT	Abbreviated impulsiveness scale: produces a continuous score
Autonomy	C/Y	SDT	Subscale of the basic needs satisfaction in general scale: produces a continuous score
Competence	C/Y	SDT	Subscale of the basic needs satisfaction in general scale: produces a continuous score
Relatedness	C/Y	SDT	Subscale of the basic needs satisfaction in general scale: produces a continuous score
Self-efficacy for physical activity	Y	SCT^d^	Self-efficacy for physical activity: produces a continuous score
Self-efficacy for healthy eating	Y	SCT	Self-efficacy to make healthy food choices: produces a continuous score

^a^C/Y: caregiver/youth.

^b^N/A: not applicable.

^c^SDT: self-determination theory.

^d^SCT: social cognitive theory.

### Secondary Outcome: Establish Costs Associated With Implementation of Mobile Health Components When Delivered With the Brenner Families in Training Program

Economic costs of delivery (ie, resource use) associated with implementing the two conditions are collected over the duration of the program. These costs allow for calculation of the full economic cost of delivering each condition, which includes direct, indirect, and opportunity costs. Examples of these may include utilization of supplies and materials (eg, the printing of materials for participants), training costs (eg, hourly wages for employees), costs associated with the actual delivery of the mHealth components, and opportunity costs (eg, volunteer time, donated materials). Net costs associated with delivering the mHealth strategies will be calculated by subtracting the costs for Brenner FIT from the costs of Brenner mFIT. Costs related to the evaluation of these strategies/conditions are excluded to capture the true economic cost of replicating the strategies across other clinics. Of the ways to express cost, one is cost per child enrolled. The more money caregivers pay for enrollment, the lower the likelihood of participation, especially when patients are from lower socioeconomic categories. Willingness and the ability to pay are assessed, as demonstrated in previous work examining the cost-effectiveness of interventions in youth [49]. However, for the proposed study, there are no additional costs for participants beyond those associated with the standard Brenner FIT program (eg, co-pays).

### Analysis Plan for Pilot Data

To inform future effectiveness of the trial, a quantitative data analysis strategy is developed as part of the proposed study to provide parameter estimates for future power calculations. For the pilot data to be collected in this study, the analysis relies on a mixed linear model to accommodate repeated assessments of BMI. This approach is anticipated to be at least as powerful as the two-sample *t* test. At each follow-up visit, the change in BMI z-score is calculated as the difference between the current and baseline z-scores. However, as some studies suggest using variations of relative BMI [50,51] or BMI for longitudinal data [52], and there continues to be a debate about the best measure to use to detect change particularly in children greater than the 97th percentile for BMI [53,54], the study group assesses the BMI, BMI z-score, and percent over 95th percentile BMI for youths with obesity [55] and secondary outcomes. These are modeled as the dependent variable, with the baseline value, assessment time point (in weeks), treatment group, and treatment by visit interaction modeled as fixed effects and subject as a random effect, with an unstructured variance-covariance matrix. Contrasts are used to test the difference between treatment groups at each visit, and the contrast for the 6-month visit forms the primary effectiveness test. Age, race, ethnicity, and sex are included in the model. Restricted maximum likelihood estimation is used to incorporate missing data under a missing at random assumption. Model assumptions are evaluated using standard diagnostics. Transformations and nonparametric alternatives are considered as needed. Sensitivity analyses to examine the impact of missing data mechanisms on our results are performed using a combination of multiple imputations, pattern mixture, and inverse probability weighting analyses, as appropriate.

Although sex as a biological variable is not believed to play a substantial role in the weight-related behaviors of adolescents, gender as a social variable is salient to this study, as girls, for example, display different age-related patterns of physical activity than boys [56], lower overall physical activity [57], and greater declines in physical activity during early adolescence (8-14 years) [58]. Thus, the team considers gender in the intervention components and in all analyses via the inclusion of gender as a dichotomous predictor and potential moderating variable.

#### Trial Sample Size

Sample size was determined relying on guidance from Eldridge et al [59] and Whitehead et al [60]. Specifically, we based our sample on a feasibility outcome (retention) and the ability to estimate parameters that will inform future trial power calculations. Given a sample size of 40 (per group), there is an anticipated range of possible precision for the retention probability estimates (CIs) as follows: a true retention rate of 60% yields a 95% CI of 55% to 75%; for a retention rate of 90%, the CI is 81% to 99%. The observed CIs provide a plausible range for the true retention rate in our population and trial conditions. If the CIs exclude values <80%, this would be an acceptable retention rate to plan a large-scale trial of Brenner *m*FIT; regardless, these estimates (CIs) are weighed heavily and, together with many other components of the trial experience, are used to determine overall acceptability and feasibility, and along with estimates of effect size, these estimates will assist with the design aspects of any future trial.

#### Scalability

This study will be informed by emerging literature on the scalability of health promotion programs [61,62]. Specifically, the several scalability considerations identified by Milat et al [61] are evaluated, such as (1) reach and adoption, (2) organizational resources required (including costs), (3) intervention delivery (eg, acceptability, fidelity), (4) contextual factors (eg, interaction of the intervention with organizational contexts), and (5) evaluation approach. For example, we will characterize the features of the clinic and its operations (eg, equipment, staffing, costs, reimbursement) to identify areas of potential variability to inform future rollout. The data collected provide answers to crucial questions suggested by Klingner et al [63]: (1) Under what conditions and with whom does Brenner *m*FIT work? (2) What is necessary to support clinical staff implementation of Brenner *m*FIT? (3) What is necessary to enhance the capacity of clinics to support staff in implementing Brenner *m*FIT under different conditions? (4) What is necessary to support broad, deep, sustained implementation of Brenner *m*FIT? This information is crucial in helping us to scale the intervention.

## Results

This study was approved by the Institutional Review Board in July 2019, funded in August 2019, and will commence enrollment in April 2020. The results of the study are expected to be published in the fall/winter of 2021.

## Discussion

### Study Goal

This study seeks to establish an effective way to boost adolescent weight loss by implementing a suite of mHealth tools in conjunction with an established pediatric weight loss clinic, Brenner FIT. The design is based on a strong theoretical background from the social cognitive theory and self-determination theory, which have been shown to be influential in adolescent weight loss. All the mobile materials are developed to be inclusive of adolescents/caregivers from different backgrounds and accessible through multiple ways. The study is designed to test the acceptability and feasibility of the mHealth intervention and compare it with the standard treatment of Brenner FIT alone. The study design attempts to minimize potential pitfalls and limitations that have plagued prior research in this realm. The results have the potential to change the current approach and clinical methodology to address adolescent weight loss through mobile technology and apps in a potentially cost-effective fashion.

### Study Limitations and Addressing Potential Concerns

Although all efforts have been made to minimize threats to validity and other potential pitfalls, a few limitations still exist related to the reduction of bias and contamination. One of the limitations is the inability to mask the conditions from the clinical staff or their assignment from the participants as it represents a potential threat to internal validity. However, the independence of the research staff from the clinical staff should protect against any expectancy effects biasing the data. Steps to prevent bias are outlined next. First, all assessments and analyses are conducted by the research staff or clinical administrative staff who are not involved in patient care and who are blinded to participant allocation. Second, data that are not normally collected during routine clinical practice will not be shared with the clinical staff who are in direct contact with patients. For example, although the clinical staff working with dyads randomized to the Brenner mFIT condition receive personalized reports based on mobile self-reporting of food consumption and physical activity, they do not receive data from the 24-hour dietary recalls, research accelerometers, or any questionnaires not normally administered as these tools are rarely available to the clinical staff in standard practice. Lastly, we will monitor the number and duration of sessions to ensure that the clinical staff are not scheduling more or longer sessions with intervention participants.

In addition, the baseline data collection concludes before group assignment is made, which will help to ensure that reactivity (if any) to being assigned to the intervention group has time to dissipate before the 3-month follow-up assessments. Meeting recruitment goals is often a concern with any clinical trial involving a finite population. However, the Brenner FIT program sees a large number of families per year that should meet the criteria, and referrals are increasing from within the Family Medicine and Pediatrics clinics of the WFBMC through targeted advertising. This increases the number of people eligible to join the proposed study.

Although participants will be recruited from and treated within the same clinic, group activities or classes are separated based on group assignment. The mobile-enabled website is password-protected, making it unlikely that the control group could access it. As we cannot blind the clinical staff to group assignment, we assess contamination via the participant report (posttest) to control for potential contamination.

### Conclusions

The ImPACT study attempts to improve on adolescent weight loss by supplementing a weight loss clinic curriculum with an mHealth curriculum. The study not only develops the extra curriculum but subsequently compares the combined treatment (Brenner FIT + *m*Health) with the standard of care (Brenner FIT) in a randomized controlled trial to assess feasibility and acceptability of the intervention. The results can help inform future research and improve clinically meaningful weight loss in adolescents.

## Appendix

Multimedia Appendix 1 National Institutes of Health Peer Review Comments.

## Abbreviations

ASA24: automated self-administered 24-hour
Brenner FIT: Brenner families in training
Brenner mFIT: Brenner FIT + mHealth
mHealth: mobile health
WFBMC: Wake Forest Baptist Medical Center
