# Supplementary material for: A Clinical Trial to Increase Self-Monitoring of Physical Activity and Eating Behaviors Among Adolescents: Protocol for the ImPACT Feasibility Study
Source: JMIR Res Protoc. 2020 Jun 5;9(6):e18098. doi: 10.2196/18098 (PMC7305562; doi:10.2196/18098)
Supplement: Multimedia Appendix 1 [file resprot_v9i6e18098_app1.pdf]

SUMMARY STATEMENT
(Privileged Communication)

PROGRAM CONTACT:
Voula Osganian
301-827-6939
voula.osganian@nih.gov

Release Date: **02/21/2019**
Revised Date: **02/21/2019**

Application Number: **1 R34 DK119815-01A1**

Principal Investigator

MOORE, JUSTIN BRIAN

Applicant Organization: WAKE FOREST UNIVERSITY HEALTH SCIENCES

Review Group: **ZDK1 GRB-1 (M1)**
National Institute of Diabetes and Digestive and Kidney Diseases Special Emphasis Panel
Natural Experiments and Pragmatic Research

Meeting Date: **02/11/2019**
Council: **MAY 2019**
Requested Start: **08/01/2019**
RFA/PA: **PAR18-924**
PCC: **NVO PCOB**

Project Title: **IMPACT: Increased Monitoring of Physical Activity and Calories with Technology**

SRG Action: **Impact Score:29**
Next Steps: **Visit https://grants.nih.gov/grants/next_steps.htm**
Human Subjects: 30-Human subjects involved - Certified, no SRG concerns
Animal Subjects: 10-No live vertebrate animals involved for competing appl.
Gender: 1A-Both genders, scientifically acceptable
Minority: 1A-Minorities and non-minorities, scientifically acceptable
Children: 1A-Both Children and Adults, scientifically acceptable

Project Year	Direct Costs Requested	Estimated Total Cost
1	150,000	238,153
2	150,000	238,153
TOTAL	300,000	476,306

ADMINISTRATIVE BUDGET NOTE: The budget shown is the requested budget and has not been adjusted to reflect any recommendations made by reviewers. If an award is planned, the costs will be calculated by Institute grants management staff based on the recommendations outlined below in the COMMITTEE BUDGET RECOMMENDATIONS section.
ADMINISTRATIVE NOTE

1R34DK119815-01A1 Moore, Justin

SCIENTIFIC REVIEW OFFICER'S NOTES

RESUME AND SUMMARY OF DISCUSSION: This resubmission application has been submitted by Dr. Justin Brian Moore on behalf of Wake Forest University in response to Program Announcement PAR-18-924, " Planning Grants for Pragmatic Research in Healthcare Settings to Improve Diabetes and Obesity Prevention and Care (R34)." The proposed study will evaluate the addition of an mHealth intervention to a pediatric weight management program. Previously identified strengths of the application remain, including the focus on obesity in teens, as there is a continued lack of knowledge related to the mechanism of behavior change in this population; the theory-driven approach based on the investigators' past work; inclusion of feasibility, fidelity, and acceptability as measures; and the investigative team and environment. Most prior critiques were well addressed. Additional strengths include measurement of retention in the study population. Concern related to the lack of generalizability due to the extraordinary environment of the Brenner FIT program remains; reviewers noted that future studies in less well-resourced environments would be critical. Overall, the application was judged to be excellent to outstanding.

DESCRIPTION (provided by applicant): Since severe obesity in youth (BMI for age ≥120th percentile) has been steadily increasing, intensive clinical intervention is necessitated, which is often delivered in specialized pediatric obesity clinics. Since the home environment and parental behavioral modeling are two of the strongest predictors of child weight loss during behavioral weight loss interventions, a family-based treatment approach is warranted. This strategy has been moderately successful in our existing, evidence-based pediatric weight management program (Brenner FIT), but since programs such as Brenner FIT rely on face-to-face delivery of programmatic elements, the dose delivered is limited by the time constraints experienced by families enrolled in treatment. Therefore, the purpose of this study is to refine and pilot a tailored, mobile (mHealth) component to augment an existing, evidence-based pediatric weight management program (Brenner FIT) to determine acceptability from a patient and clinical staff perspective, feasibility, and economic costs relative to the established weight management protocol alone [i.e., Brenner FIT vs. Brenner FIT + mHealth (Brenner mFIT)]. Primary Aim 1: Finalize an intervention for adolescent youth with obesity, consisting of podcasts/videos and electronic self-monitoring, to promote reduced caloric intake and increase physical activity. Primary Aim 2: Pilot the intervention in dyads (n = 40) recruited from a pediatric weight loss clinic, to establish acceptability and feasibility of the intervention relative to standard care (n = 40). Secondary Aim: To establish costs associated with development and implementation of mHealth components when delivered with the Brenner FIT program. Study design: For this project, we will randomize 80 youth with obesity (13 – 18yrs) and a caregiver (dyads) to the Brenner FIT condition or the Brenner mFIT condition. All participants will complete baseline measures prior to randomization, and at three and six months. All youth participants will receive a commercially available activity monitor. Caregivers will receive podcasts with a story about a caregiver supporting weight loss in a child by providing healthy foods/activities for his/her family, including healthy eating and physical activity information. Children will receive animated videos that contain healthy eating and physical activity messaging, with an engaging story of a child losing weight. All participants will have access to a website and mobile apps where they will track weight, diet, and physical activity for themselves (youth) or their child (parents). Based on their reports of weight, eating, and physical activity, the messaging received from clinical staff by the families will be individually tailored to promote healthy behaviors and overcome perceived barriers. The proposed research is innovative in that it explicitly incorporates theory into the intervention and evaluation components of the project and builds upon an existing literature on mHealth interventions that use mobile technology.

PUBLIC HEALTH RELEVANCE

This project has great public health value in that it addresses the significant need of clinicians for tools to augment current obesity treatment programs. This project will lead to the development of new tools (e.g., podcasts, videos) and a protocol for the use of commercially available tools which are or will be

available to clinicians and families. This is relevant, because current clinical interventions/treatments for youth with obesity have been only marginally successful despite being resource intensive, but if lower cost, higher reach tools can be developed to leverage existing technology, more youth could be reached in a cost-effective manner.

CRITIQUES: The written critiques of individual reviewers are provided in essentially unedited form below. These critiques were prepared prior to the meeting and may not have been revised afterwards. The "Resume and Summary of Discussion" above summarizes the final opinions of the committee.

CRITIQUE 1

Significance: 3

Investigator(s): 1

Innovation: 2

Approach: 3

Environment: 1

Overall Impact:

For this R34 application, the investigators propose to develop the Brenner FIT mhealth intervention components and conduct a pilot study comparing the feasibility and efficacy of this intervention to the standard Brenner FIT program in adolescents and their caretakers. The standard program is a multidisciplinary, comprehensive pediatric weight management program consisting of in-person individual and group-based visits with multidisciplinary staff and paper-based diet and physical activity tracking. The mHealth intervention would add several components including animated video stories, podcasts, social media, and app-based physical activity and dietary tracking. The investigators anticipate that the mhealth components will increase adherence and engagement in the weight management program. Overall, this study addresses an important problem in innovative ways. The investigative team is quite strong and has ample experience conducting weight management studies in real-world settings using mhealth components. Critiques of the study include that Brenner FIT program is well-resourced and programs like this are relatively rare across the country; therefore, testing the mHealth components in this setting may not be generalizable to lower-resourced settings. The process of using clinic staff to review online self-monitoring data and providing texting and email feedback in not automated, potentially providing increased staff burden and variable implementation. Finally, the brief timeframe for Aim 1 does not allow for iterative improvements and testing to work out bugs prior to doing the RCT in Aim 2.

Strengths

- This application address obesity in adolescents, a significant public health concern
- Mobile technologies can improve adherence to treatment and are acceptable in this population.
- This study addresses the often-poor methodological quality of prior adolescent obesity treatment interventions by using theory-based design, including caregivers, and improving use of technology
- Using the IDEAS framework to design the mhealth tool

Weaknesses

- Brenner FIT is a strong, well-resourced, comprehensive, pediatric weight management clinic. These resources are relatively rare throughout the country and may not be generalizable to other settings that do not have these resources. If found to be efficacious, the investigators may in the future want to test the mhealth pieces of the program in a lower-resource setting.

2. Investigators:

Strengths

- Dr. Moore has a strong record of leading obesity and physical activity interventions
- Interdisciplinary team with expertise in pediatric obesity, intervention dissemination, biostatistics, cost analyses
- Dr. Miller and Dr. DeLeo have expertise in digital health and integrating mhealth into clinical practice
- Team has a record of collaboration

Weaknesses

- None noted

3. Innovation:

Strengths

- M health tool will be designed to facilitate communication between adolescents and caregivers
- The healthcare providers will use self-monitoring data to provide tailored feedback via texts and emails.
- The use of podcasting and social media is innovative and has been successfully implemented in the investigators' prior studies. Animated videos could engage adolescents in a meaningful way.
- The mhealth components are informed by a Theory-based approach

Weaknesses

- The process using of clinic staff to review online self-monitoring data and providing texting and email feedback in not automated, potentially providing increased staff burden and variable implementation

4. Approach

Strengths

- Participants will be recruited and randomized from Brenner FIT, an existing pediatric weight management program
- All components of the interventions will be delivered by clinical staff
- While participants cannot be blinded, assessments will occur by research staff not involved with intervention delivery
- The materials and videos will be designed to be culturally informed to serve diverse patients
- Investigators will measure theoretical constructs from Social Cognitive and Self-Determination theories
- Feasibility measures will include "retention" of attending >75% of sessions in 6 months—this would be a great outcome in itself to compare between the 2 arms. The feasibility measures will include time staff spent on training and technical support related to mhealth components.
- They will track implementation fidelity:
- The investigators propose a cost analysis as a secondary aim and will work with health economist

Weaknesses

- There is a rapid timeframe for Aim 1 with no description on whether the investigators will pilot testing materials with cognitive interviews/conducting usability testing or even demonstration to

prior focus groups and interviewees prior to recruiting the 80 dyads in Aim 2. As patients are recruited and go through the mhealth intervention, there will be bugs and issues. There is no mention as to whether and how there will be iterative development along the way to address potential issues.

- Brenner FIT sees 200 families per year (if half are eligible, this assumes that the majority of eligible patients will agree to participate in this study). Even though the investigators will help increase referrals to the Brenner Fit program, an estimation of how many more families they would need to recruit (based on expected eligibility and enrollment) would be helpful.
- While it is understandable to have English materials only for a pilot given limited resources, the investigators do not state intent or plans to translate into Spanish in the future.
- The cost analysis should include more detail about anticipated costs to both staff and patients.
- The dissemination plan does not discuss dissemination of the mhealth toolkit-this is mentioned in the significance section.

5. Environment:

Strengths

- Brenner's children's hospital has a program called Brenner FIT, a pediatric weight management program where participants will be recruited and that will serve as the control condition
- The Children's Hospital serves a diverse patient population with 40% Black and 45% white
- Wake Forest has a strong academic mission and environment
- The University of South Carolina has the Arnold School of Public Health

Weaknesses

- None noted

Study Timeline:

Strengths

- The recruitment timeline seems adequate for Aim 2

Weaknesses

- Six months seems quick to develop the mhealth components including animated videos and podcasts and there is little time to pilot test or

Milestone Plan:

Strengths

- Investigators have meaningfully laid out the recruitment targets

Weaknesses

- None noted

Protections for Human Subjects:

Acceptable risks/adequate projections

Since the participants will receive texts and emails from the intervention staff as part of this intervention, there should be mention of how this will be done in a way that is HIPPA compliant and secure. This is a low risk study with adequate protection against risk.

Data and Safety Monitoring Plan (Applicable for Clinical Trials Only):

Acceptable

While DSMB is not needed, there will be a scientific review board.

Inclusion of Women, Minorities and Children Applicable Only for Human Subjects research.

- Sex/Gender: inclusion justified scientifically
- Race/Ethnicity: inclusion justified scientifically
- For NIH-Defined Phase III trials, Plans for valid design and analysis:
- Inclusion/Exclusion of Children under 18: inclusion justified scientifically
- Will have low numbers of latinx participants since will not doing intervention in Spanish

Vertebrate Animals

- NA

Biohazards:

- NA

Resubmission:

- Investigators adequately addressed prior reviewer concerns about dissemination, sample size, recruitment goals, potential for contamination, levels of cell phone ownership, timeline, and collaboration among research team.
- 0.6 FTE for RA still seem low for the amount of time for recruitment needed
- Generalizability was addressed by stating that there are 85 similar programs to Brenner FIT in the US that could potentially use the mhealth component to increase engagement and adherence to their programs. This still seems somewhat low.

Budget and Period of Support:

recommend as requested

Recommended budget modifications or possible overlap identified:

- May want to consider more time for RA

CRITIQUE 2

Significance: 3
Investigator(s): 2
Innovation: 2
Approach: 3
Environment: 1

Overall Impact:

This team proposes to augment an existing clinical obesity program (Brenner Fit) with an mHealth component to improve weight loss outcomes among adolescents. If successful, this work can inform future strategies in clinical care of Stage 3 and 4 clinics for obesity. However, there will be limited generalizability to primary care settings. Also, the Brenner mHealth group receives a lot of “intervention” beyond that of the Brenner FIT group and there is no attention control to account for that difference in dose.

1. Significance

Strengths

- Well-stated scientific rationale
- Theory-based intervention development
- If successful, the proposed mHealth approach can inform strategies in healthcare practice to improve retention and potentially weight loss outcomes among adolescents in a weight loss clinic.

Weaknesses

- Limited generalizability due to the implementation into Brenner Fit. Referral into this clinic suggests a level of care and engagement beyond primary care which may introduce some selection bias. While there are several other similar clinics nationally, integration into primary care would yield the best opportunity for scalability.

2. Investigators:

Strengths

- Dr. Moore, PI, is experienced in the promotion of physical activity and healthy eating in youth and adults with a track record of publishing work in this area.
- Dr. DeLeo brings expertise in developing technology for use in health care.
- Dr. Heboyan as a consultant strengthens the cost-effectiveness aspect of the application.

Weaknesses

- No effort for consultant?

3. Innovation:

Strengths

- The integration of behavioral theory and use of a systematic framework in the development process.

Weaknesses

- mHealth to supplement clinical care is less novel at this point.

4. Approach

Strengths

- The approach is informed by preliminary and formative work conducted by the research team.
- Existing infrastructure of Brenner FIT
- Plans for diversity and cultural competency in the development team

Weaknesses

- In the description of the design it refers to n=80 dyads per group, but in most other places it appears that groups will contain 40 dyads each. I agree with the proposal of 40 dyads per group as 80 per group may not be feasible for this study.
- The identification of recruits may cause additional burden on the Brenner FIT staff. While there is some mention of a research team member, this would also make sustainability challenging after the intervention period.
- Lack of attention control

5. Environment:

Strengths

- Wake Forest Baptist Medical Center offers a strong environment to conduct the proposed work.
- Brenner Children’s Hospital is home to Brenner FIT and makes an ideal setting to test Brenner mFIT.
- Arnold School of Public Health at USC has the resources to collaborate successfully on this project including the Children’s Physical Activity Research Group.

Weaknesses

- None noted.

Study Timeline:

Strengths

- The application will utilize the medical record to pre-identify potential participants.
- Adequate time built in for development at the beginning of the study.

Weaknesses

- No contingency plan for recruitment challenges.

Milestone Plan:

Strengths

- Detailed description of recruitment targets throughout the study period.

Weaknesses

- None noted.

Protections for Human Subjects:

- Adequate

Data and Safety Monitoring Plan (Applicable for Clinical Trials Only):

Adequate

Inclusion of Women, Minorities and Children Applicable Only for Human Subjects research.

- Sex/Gender: scientifically justified
- Race/Ethnicity: scientifically justified
- For NIH-Defined Phase III trials, Plans for valid design and analysis:
- Inclusion/Exclusion of Children under 18: scientifically justified

Vertebrate Animals

- Not applicable

Biohazards:

- Not applicable

Renewal:

Budget and Period of Support:

Recommended budget modifications or possible overlap identified:

- It is unclear whether funds are being paid to the consultant.

CRITIQUE 3

Significance: 3
Investigator(s): 2
Innovation: 3
Approach: 3
Environment: 1

Overall Impact:

The project addresses an important public health challenge and has the potential to augment the benefits of a specialty adolescent weight loss program. The timeline is quite ambitious (though less so than in the original application). Also, the fact that the mHealth application exists only coupled to a robust specialty clinic may limit the ability to disseminate widely even if successful.

1. Significance

Strengths

- Uses theory-based approach to address adolescent obesity.
- mHealth approach potentially easy to scale if successful.
- Aims to address high dropout rates observed in existing programs.

Weaknesses

- Conducted via referral hospital program specialized in pediatric obesity which may reduce generalizability.
- No mHealth only group which may be the only feasible intervention for some patients in need of weight loss support.

2. Investigators:

Strengths

- Investigative team has the necessary skills and expertise.
- Multi-disciplinary
- Relevant prior trial experience.

Weaknesses

- None noted.

3. Innovation:

Strengths

- Multimodality mHealth approach tailored to needs of adolescents.
- Use of theory in development process.

Weaknesses

- Mobile application for weight loss per se isn't innovative but there are innovative aspects to how this will be done.

4. Approach

Strengths

- They have already completed a 20 family formative study.
- Diverse pool of potential participants.
- Blinded outcomes assessors.

Weaknesses

- No mHealth only group.
- Timeline appears more realistic than prior submission but is still tight. In particular, production of videos within several month time frame may be unrealistic.
- Study is labeled as NIH phase 3 clinical trial but this doesn't seem to fit.

5. Environment:

Strengths

- Presence of strong pediatric weight management program.

Weaknesses

- None noted.

Study Timeline:

Strengths

- Well laid out.

Weaknesses

- May be a bit tight for podcast development.

Milestone Plan:

Strengths

- Will allow for tracking performance.

Weaknesses

- Completion of content for Brenner mFIT could be included as milestone.

Protections for Human Subjects:

Acceptable Risks and/or Adequate Protections

Data and Safety Monitoring Plan (Applicable for Clinical Trials Only):

Acceptable

Inclusion of Women, Minorities and Children Applicable Only for Human Subjects research.

- Sex/Gender: Distribution justified scientifically
- Race/Ethnicity: Distribution justified scientifically
- For NIH-Defined Phase III trials, Plans for valid design and analysis: Scientifically unacceptable

- Inclusion/Exclusion of Children under 18:
- Not clear that this meets criteria for Phase 3 trial.

Vertebrate Animals

Not Applicable (No Vertebrate Animals)

Biohazards:

Not Applicable (No Biohazards)

Resubmission:

- Addressed multiple concerns raised in original review. Appropriately reduced recruitment target.

Budget and Period of Support:

Recommend as Requested

Recommended budget modifications or possible overlap identified:

Resource Sharing Plans:

Acceptable

CRITIQUE 4

Significance: 2
Investigator(s): 2
Innovation: 1
Approach: 3
Environment: 2

Overall Impact:

The proposed research aims to address a significant problem: obesity in teens. Brenner FIT is a theoretically-driven weight loss intervention for adolescents, which has previously shown to achieve clinically significant weight loss in this population. The development of a hybrid mHealth adaptation to this evidence-based approach is novel. Further, this pragmatic pilot/feasibility trial is well-designed; inclusion of feasibility, fidelity, and acceptability measures is a strength. Further, the planned outcome evaluation is comprehensive and the investigative team is well-suited to conduct the scope of work. The original application was rated as very good to excellent, with some areas for improvement.

Overall, it is felt that the investigators have adequately addressed the concerns related to: (1) Ambitious sample size/recruitment timeline (reduced to 80 dyads); (2) Inadequate FTE for research assistant (increased to 0.6 FTE); (3) unnamed biostatistician (Eddie Ip); (4) Investigator qualifications (well-qualified); and (5) feasibility of the development phase/Aim 1 and contingent nature of Aim 2 (development prior to study start will improve feasibility). While some potential for contamination remains given that clinical staff will see patients in both groups, the investigators indicate they will assess for this at post-test. In response to concerns about generalizability, the investigators make the claim that >85 programs *similar* to Brenner FIT exist nationwide – however, a minor remaining weakness is that it is unclear if these settings are pediatric obesity clinics, or if these programs are also feasible in primary care clinics – i.e., a much more common venue, where much of childhood obesity is encountered.

While some uncertainty remains about if/how engagement with the mHealth intervention will be maintained, this is an important question that the proposed research can seek to answer. Overall, the

remaining concerns are very minor and do not detract significantly from the proposed research's very high potential for impact.

THE FOLLOWING SECTIONS WERE PREPARED BY THE SCIENTIFIC REVIEW OFFICER TO SUMMARIZE THE OUTCOME OF DISCUSSIONS OF THE REVIEW COMMITTEE, OR REVIEWERS' WRITTEN CRITIQUES, ON THE FOLLOWING ISSUES:

PROTECTION OF HUMAN SUBJECTS: ACCEPTABLE

INCLUSION OF WOMEN PLAN: ACCEPTABLE

INCLUSION OF MINORITIES PLAN: ACCEPTABLE

INCLUSION OF CHILDREN PLAN: ACCEPTABLE

SCIENTIFIC REVIEW OFFICER'S NOTES:

The study is classified as an NIH Phase 3 clinical trial in the application, but reviewers noted that this classification does not seem to be correct.

COMMITTEE BUDGET RECOMMENDATIONS: The budget was recommended as requested.

Footnotes for 1 R34 DK119815-01A1; PI Name: Moore, Justin Brian

NIH has modified its policy regarding the receipt of resubmissions (amended applications). See Guide Notice NOT-OD-14-074 at http://grants.nih.gov/grants/guide/notice-files/NOT-OD-14-074.html. The impact/priority score is calculated after discussion of an application by averaging the overall scores (1-9) given by all voting reviewers on the committee and multiplying by 10. The criterion scores are submitted prior to the meeting by the individual reviewers assigned to an application, and are not discussed specifically at the review meeting or calculated into the overall impact score. Some applications also receive a percentile ranking. For details on the review process, see http://grants.nih.gov/grants/peer_review_process.htm#scoring.

MEETING ROSTER

The roster for this review meeting is displayed as an aggregated roster that includes reviewers from multiple DK Special Emphasis Panels of the Diabetes and Obesity Policy and Healthcare for the 2019/05 council round.
This roster for DK is available at:
http://public.era.nih.gov/pubroster/Reports?DOCTYPE=SEP&DESFORMAT=PDF&AGENDA_SEQ_NUM_P=358869
